# Efficacy of targeted AKT inhibition in genetically engineered mouse models of *PTEN*-deficient prostate cancer

**DOI:** 10.18632/oncotarget.7557

**Published:** 2016-02-21

**Authors:** Marco A. De Velasco, Yurie Kura, Kazuhiro Yoshikawa, Kazuto Nishio, Barry R. Davies, Hirotsugu Uemura

**Affiliations:** ^1^ Department of Urology, Kinki University Faculty of Medicine, Osaka-Sayama, Osaka, Japan; ^2^ Department of Genome Biology, Kinki University Faculty of Medicine, Osaka-Sayama, Osaka, Japan; ^3^ Division of Advanced Research Promotion Institute of Comprehensive Medical Research, Aichi Medical University, Nagakute, Aichi, Japan; ^4^ Oncology iMED, AstraZeneca, Alderley Park, Macclesfield, UK

**Keywords:** prostate cancer, AKT inhibitor, targeted therapy, preclinical model, PTEN

## Abstract

The PI3K/AKT pathway is frequently altered in advanced human prostate cancer mainly through the loss of functional *PTEN*, and presents as potential target for personalized therapy. Our aim was to determine the therapeutic potential of the pan-AKT inhibitor, AZD5363, in *PTEN*-deficient prostate cancer. Here we used a genetically engineered mouse (GEM) model of *PTEN*-deficient prostate cancer to evaluate the *in vivo* pharmacodynamic and antitumor activity of AZD5363 in castration-naïve and castration-resistant prostate cancer. An additional GEM model, based on the concomitant inactivation of *PTEN* and *Trp53 (P53)*, was established as an aggressive model of advanced prostate cancer and was used to further evaluate clinically relevant endpoints after treatment with AZD5363. *In vivo* pharmacodynamic studies demonstrated that AZD5363 effectively inhibited downstream targets of AKT. AZD5363 monotherapy significantly reduced growth of tumors in castration-naïve and castration-resistant models of *PTEN*-deficient prostate cancer. More importantly, AZD5363 significantly delayed tumor growth and improved overall survival and progression-free survival in *PTEN/P53* double knockout mice. Our findings demonstrate that AZD5363 is effective against GEM models of *PTEN*-deficient prostate cancer and provide lines of evidence to support further investigation into the development of treatment strategies targeting AKT for the treatment of *PTEN*-deficient prostate cancer.

## INTRODUCTION

Androgen-deprivation therapy remains the primary treatment option for patients with metastatic prostate cancer. However, most of these individuals will inevitably develop resistance and progress to a form of the disease referred to as castration-resistant prostate cancer (CRPC). Despite therapeutic improvements with recently developed second-generation antiandrogens, affected patients fail to maintain treatment response and eventually develop secondary resistance [1-3]. Moreover, CRPC is a highly heterogeneous disease with multiple underlying mechanisms driving its emergence, progression and survival. Recently, a number of studies have focused on characterizing the molecular landscape of advanced prostate cancer to identify networks with potentially druggable targets that may aid in the development of better treatment strategies [4-6].

Identification of potential targets has spurred the development of several novel compounds. Yet, high attrition rates are seen, often as a result of poor predictability from traditional preclinical testing models [7]. GEM models have emerged as potentially superior models for preclinical efficacy evaluation [8]. However, as in any model, limitations do exist and the body of evidence to assess actual predictability is limited. One approach to improve preclinical predictability of novel targeting agent is to include pharmacologically realistic dosing and include clinically relevant endpoints. GEM are designed to recapitulate molecular and biological features of human cancer, and afford a number of features lacking in xenograft models, thus, making it a choice animal model to assess the preclinical efficacy of novel therapeutic compounds [9].

The PI3K/AKT pathway is a highly conserved signal transduction pathway that under normal conditions regulates cell metabolism, growth and survival during cellular stress. However, this pathway is frequently activated in human prostate cancer as a result of genetic alterations that include the biallelic loss of *PTEN* and activating mutations in AKT1 and PIK3CA/B [4-6, 10]. Activation of PI3K occurs *via* a series of upstream regulatory signals from membrane receptor tyrosine kinases (RTKs) and non-RTKs that in turn activate AKT [11]. Under normal conditions, activation of PI3K is negatively regulated by the tumor suppressor phosphatase and tensin homolog deleted on chromosome 10 (*PTEN*) [12]. AKT has become an attractive therapeutic target since it plays a key role as a central molecule that modulates a wide range of cellular processes associated with the progression of tumors such as survival, proliferation, cell cycle progression, growth, invasion, migration, and angiogenesis [13].

AZD5363 is novel pyrrolopyrimidine derivative and potent ATP-competitive inhibitor of all AKT kinases [14]. Preclinical studies have shown a correlation between the sensitivity to AZD5363 and the presence of *PIK3CA* and/or *PTEN* mutations in cultured human cancer cell lines *in vitro* and *in vivo* [14-16]. In the present study, we examined the therapeutic potential of AKT inhibition in mouse prostate cancer with altered PI3K/AKT pathway activation. Specifically, we describe the preclinical effects of AZD5363 in a series of GEM models of prostate cancer driven by the conditional inactivation of *PTEN* and *PTEN* and *Tp*53 (*P53)*.

## RESULTS

### Pharmacodynamic effects of AZD5363 in PTEN-deficient prostate cancer

**Figure 1:**
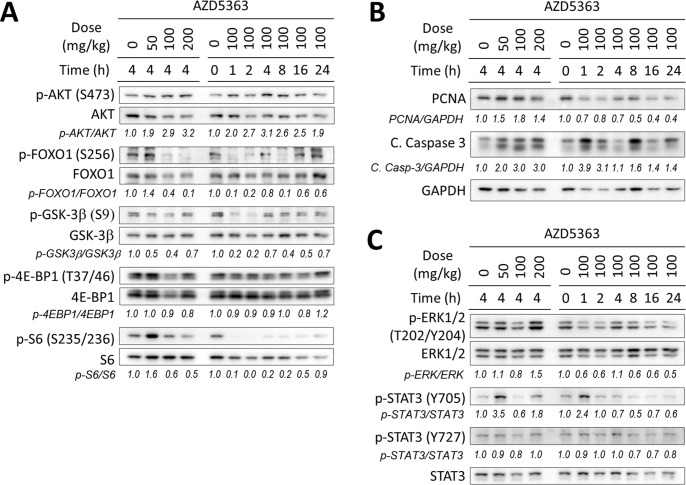
*In vivo* pharmacodynamic activity of AZD5363 in mouse *PTEN*-deficient prostate tumors Twenty-week-old *PTEN*-KO mice (*n* = 3 mice per group) bearing prostate tumors were treated with AZD5363 for the indicated dosage and times. Tumors lysates were pooled and were examined by western blot for the expression of proteins and/or phosphorylation of AKT and its downstream targets **A.**, markers of proliferation and apoptosis **B.**, and markers of the MAPK and JAK/STAT3 signaling pathway **C.** Gel densitometry was quantified with ImageJ.

Our first aim was to determine the pharmacodynamic (PD) effects of AZD5363 in a preclinical model of prostate cancer that shares similar features and genetic alterations associated with the human disease. For this we used the *PTEN*-KO mouse model in which the development of prostate tumors driven by the conditional inactivation of *PTEN* [17]. The PD activity of AZD5363 was determined by its ability to inhibit the phosphorylation AKT substrates (FOXO1, GSK3β) and downstream pathway biomarkers (4E-BP1 and S6), after a single oral dose in prostate tumor tissue. AZD5363 effectively inhibited the phosphorylation of the AKT substrates at a dose of 100 mg/kg and maximal inhibitory activity was observed within the first 2 h following administration (Figure 1A). Inhibitory activity of AZD5363 at 100 mg/kg was maintained for at least 8 h for phosphorylation of FOXO1 and S6 before returning to baseline levels. We next investigated the effects of AZD5363 on markers of cell proliferation (PNCA) and apoptosis (cleaved caspase-3) by western blot. AZD5363 decreased levels of PCNA after 16 h, and levels of cleaved caspase-3 spiked 4-fold at after 1 h, suggesting that acute inhibition of AKT signal activation modulated the suppression of cellular proliferation and induced apoptosis (Figure 1B).

The RAS/RAFMAPK and JAK/STAT3 signal pathways have been implicated with the resistance and survival of cancer cells [18, 19]. Therefore, we sought to investigate the effects of AZD5363 administration on RAS/RAFMAPK and JAK/STAT3 signaling by measuring the phosphorylation of ERK1/2, STAT3 (Y705) and STAT3 (S727). Notably, phosphorylation levels of STAT3 (Y705) spiked > 2-fold 1h after the administration of AZD5363 when administered at 100 mg/kg before decreasing below baseline levels (Figure 1C). Interestingly, levels of STAT3 (Y705) increased after 4 h when administered at 50 and 200 mg/kg. Levels of ERK phosphorylation increased after dosing 200 mg/kg AZD5363, but remained at or below baseline levels during the time course experiment when administered at 100 mg/kg.

### AZD5363 monotherapy induces therapeutic responses in mouse PTEN-deficient prostate cancer

**Figure 2:**
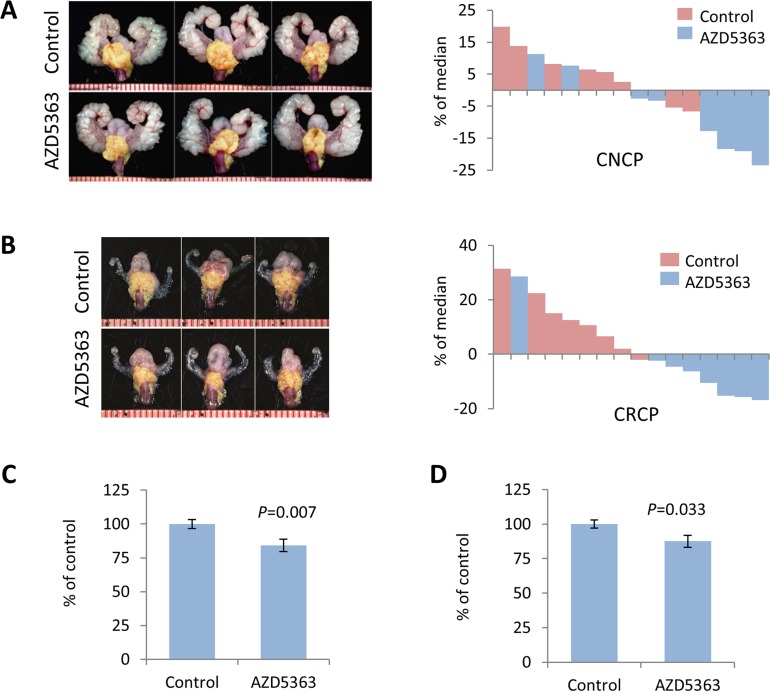
Chronic dosing of AZD5363 decreases tumor burden in *PTEN-*deficient models of prostate cancer Twenty-week-old *PTEN*-KO mice with CNPC or CRPC were randomized (*n* = 8 per group) and treated vehicle (control) or AZD5363 (100 mg/kg b.i.d.) for 4 weeks. Representative images of GUTs *en bloc* and corresponding waterfall plots of individual treatment responses for CNPC **A.**, and CRPC **B.** Prostate tumors are indicated by yellow mask. Plots of overall tumor burden measured bu tumor area for CNPC **C.**, and CRPC **D.** Values represent mean ± s.e.

**Figure 3:**
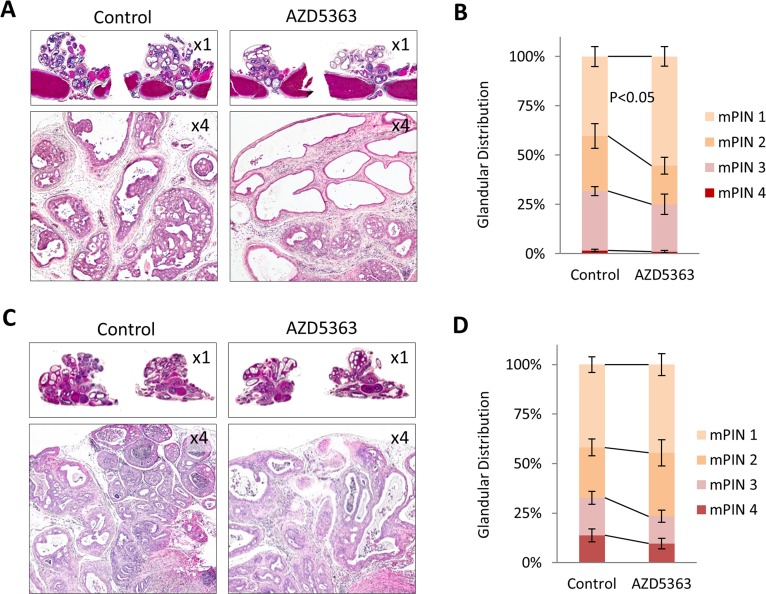
Treatment with AZD5363 reduces *PTEN-*deficient tumor progression Representative H&E stained sections of CNPC **A.**, and CRPC **C.**, from control and AZD5363 treated mice. Histopathological analysis of mPIN distribution in CNPC **B.**, (*n* = 8 mice per group) and CRPC **D.**, (*n* = 5 mice per group).

We next evaluated the antitumor activity of AZD5363 monotherapy in models of *PTEN*-deficient CNPC and CRPC. As in the PD studies, we utilized *PTEN*-KO mice and administered AZD5363 100 mg/kg b.i.d. (5 days on and 2 days off) for a period of 4 weeks (Supplementary Figure S1A). The dosage was derived from drug activity observed in the PD study and other preclinical studies [14]. Experimental endpoints were quantification of tumor burden, influence on proliferation and apoptosis, and western blot and IHC analyses of signal transduction. AZD5363 significantly reduced tumor burden in mice in both CNPC and CRPC models compared to controls (Figure 2A-2D). Histological analysis of the lateral and ventral lobes of the prostates from CNPC and CRPC mice treated with AZD5363 revealed a modest decrease in the proportion of high-grade mouse intraepithelial neoplasia (mPIN3 and mPIN4) compared to controls, however, this trend did not achieve statistical significance (Figure 3A-3D). Additionally, a tendency for an increase in the proportion of low-grade mPIN (mPIN1 and mPIN2) was observed in CNPC and CRPC after treatment with AZD5363.

**Figure 4:**
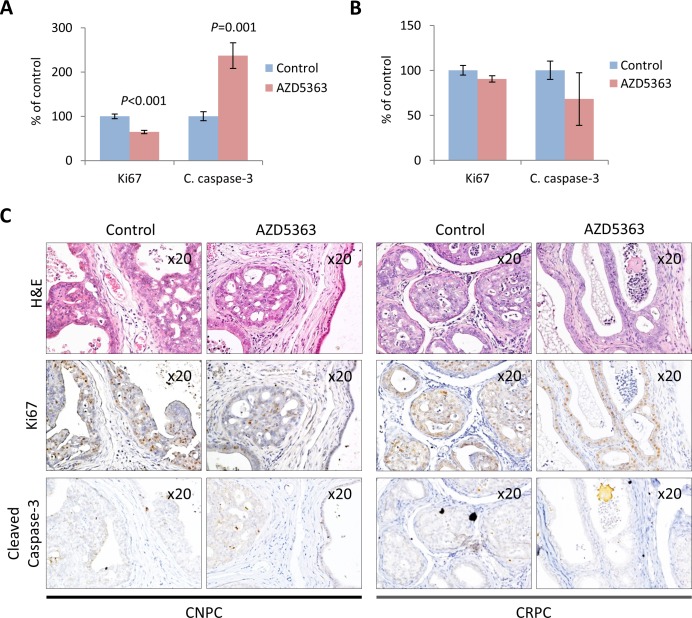
Effects of chronic AZD5363 treatment on tumor proliferation and apoptosis IHC quantification of proliferation (Ki67) and apoptosis (cleaved caspase-3) in CNPC **A.**, (*n* = 6 mice per group, and CRPC **B.**, (*n* = 5 mice per group). Values represent mean ± s.e. **C.** Representative images of H&E stained CNPC or CRPC tumor from control and AZD5363 treated mice and their corresponding serial sections immunostained with Ki67and cleaved caspase-3.

We investigated the growth inhibitory effects of AZD5363 therapy on CNPC and CRPC by measuring tumor cell proliferation and apoptosis by IHC. Statistically significant reduction of proliferation and increase in apoptosis in tumors was observed in mice treated with AZD5363 compared to controls in the CNPC model (Figure 4A, 4C). In the CRPC model, tumors from mice treated with AZD5363 revealed no significant changes in proliferation and apoptosis compared to controls (Figure 4B, 4C). A high number of inactive glands, characterized by distension and composed of a single layer of cuboidal cells with low Ki67 and cleaved caspase-3 reactivity, were observed in AZD5363-treated mice in both models (Figure 4C). The increased presence of these types of glands may also suggest a therapy-mediated senescent response to AZD5363.

**Figure 5:**
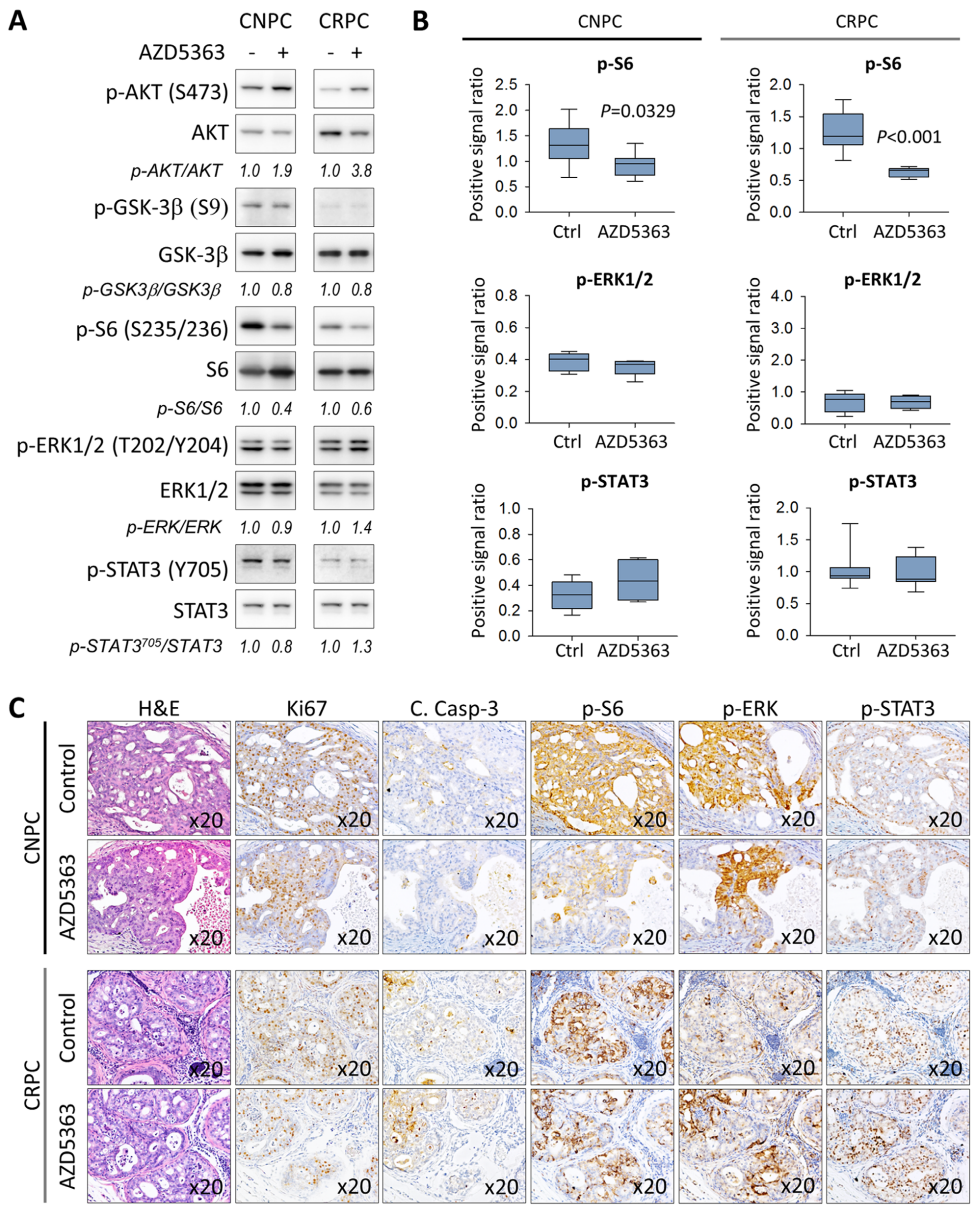
Characterization of the PI3K/AKT, MAPK, and JAK/STAT3 signaling pathways in *PTEN*-deficient prostate cancer in response to treatment with AZD5363 **A.** Tumors lysates from control and AZD5363 treated mice (*n* = 6 per group) were pooled and were examined by western blot for the expression targets for PI3K/AKT, MAPK, and JAK/STAT3 signaling. Gel densitometry was quantified with ImageJ. Lanes were run on the same gel but were noncontiguous. **B.** IHC quantification of phosphorylated S6 (S235/236), ERK (T202/Y204) and STAT3 (Y705) proteins of tissue sections corresponding to **A.** Values represent mean ± s.e. **C.**, Representative images of H&E and IHC staining patterns of Ki67, cleaved caspase-3, and phosphorylated S6 (S235/236), ERK (T202/Y204), and STAT3 (Y705) in vehicle and proliferative active regions in AZD5363 treated mice.

We next sought to investigate the effects of chronic AZD5363 therapy on the activation of the PI3/AKT signaling as well as the status of MAPK and JAK/STAT3 signal transduction. Consistent with the PD data, western blot analysis revealed that AZD5363 effectively inhibited the phosphorylation of S6 in both animal models (Figure 5A-5B). Overall, chronic dosing of AZD5363 did not appear significantly affect the phosphorylation of molecular targets of MAPK and JAK/STAT3 (Figure 5A-5B). However, IHC analysis revealed that proliferative active regions expressed high levels of both phosphorylated ERK and STAT3 despite reduced S6 activity in CNPC (Figure 5C). In CRPC, the overall expression of phosphorylated S6 was reduced but remained active in certain tumor cells which tended to also express increased phosphorylated ERK and STAT3 (Figure 5C). Overall, these findings show that AZD5363 is capable of inhibiting AKT activity thus reducing tumor growth and progression in early-stage models of CNPC and CRPC.

### Development of a prostate-specific PTEN/TP53 conditional double knockout mouse model of prostate cancer

**Figure 6:**
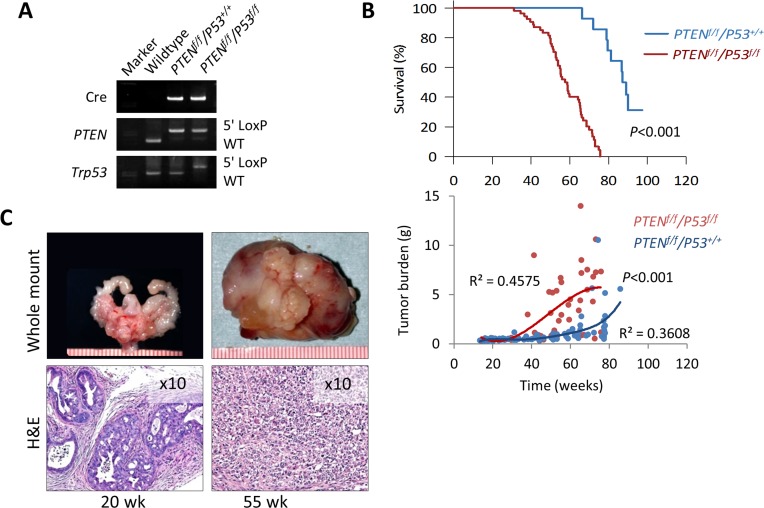
Characterization of prostate cancer development in *PTEN/P53-DKO* mice **A.** PCR confirmation of Cre-mediated recombination of *PTEN* and *P53*. **B.** Comparison of cumulative survival and prostate tumor burden (GUT weight) between *PTEN*-KO and *PTEN/P53*-DKO mice. **C.** Representative gross and histological images of early (20 w) and late-stage (55 w) prostate tumors from *PTEN/P53*-DKO mice. Ruler scale is in mm increments.

**Figure 7:**
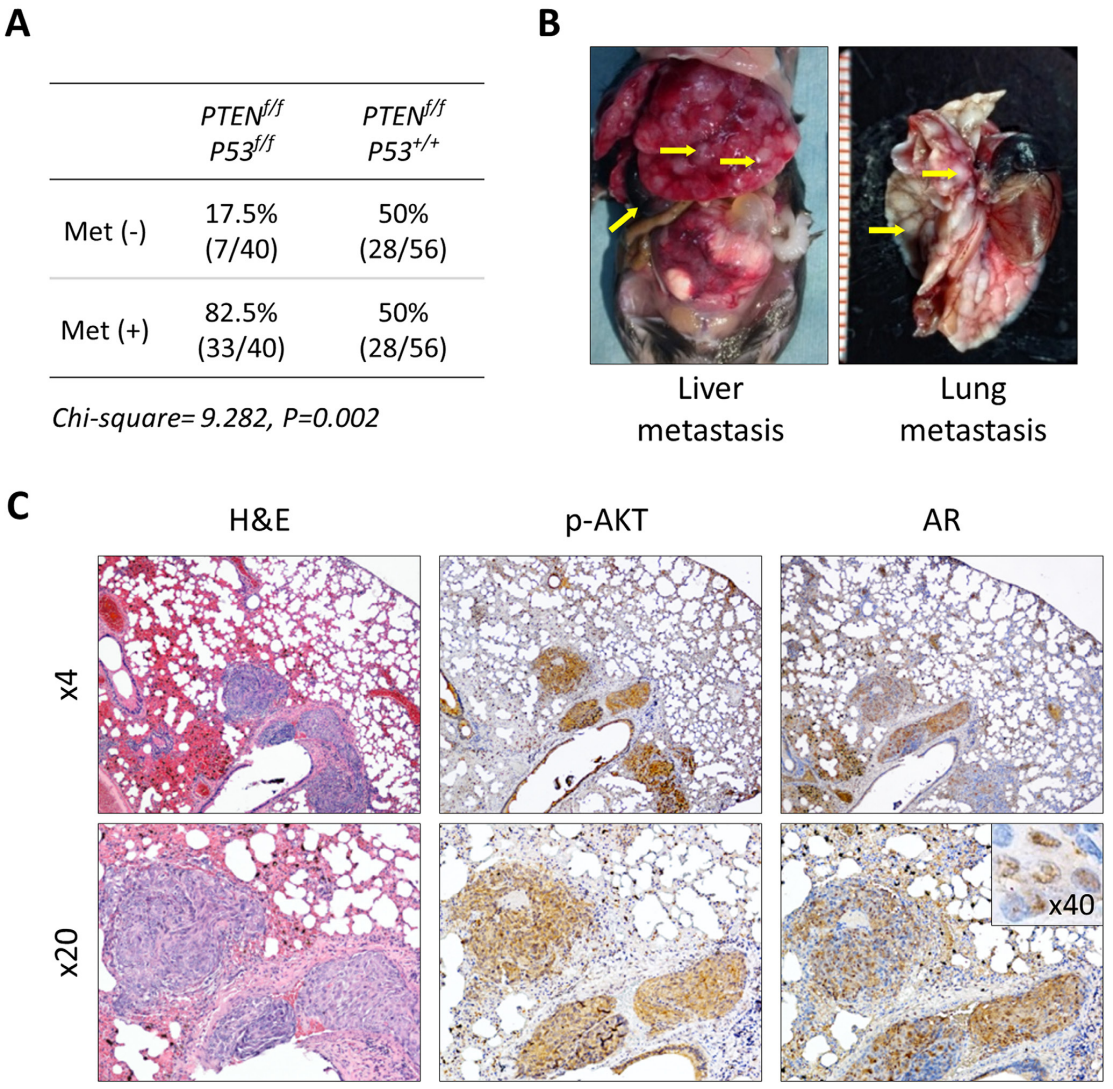
Development of metastatic prostate cancer in *PTEN/P53-DKO* mice **A.** Comparison of metastatic prostate cancer development in conditional *PTEN* and *PTEN/P53* knockout mice. **B.** Representative images of distant metastasis in *PTEN/P53*-DKO mice. **C.** Histological and IHC staining of phosphorylated AKT and AR prostate cancer metastasis growing in lung.

Short latency and consistency in tumor development has made the *PTEN*-KO mouse model a useful tool to study the direct antitumor activity of novel agents and characterize pertinent molecular mechanisms during the early stage of prostate cancer development [17, 20, 21]. At the same time, tumor growth is slow and mice tend to live a long time, making this model impractical to conduct long-term studies that are required in order to measure clinically relevant outcomes such as survival, disease progression, tumor burden, and performance status. To address this issue, we established a double knockout mouse model based on the conditional inactivation of the *PTEN* and *P53* tumor suppressor genes (Figure 6A). Inactivation of *P53* is a feature that is frequent in advanced human prostate cancer and has been shown to contribute to disease progression in animal models [22, 23]. We first examined the effect of conditional inactivation of *P53* by *PSA-Cre*. Null-*P53* did not lead to the initiation of prostate cancer in mice followed for one year. However, biallelic inactivation of both, *PTEN* and *P53* produced an aggressive phenotype characterized by significantly decreased survival compared to single gene or monoallelic gene inactivation or ether gene (Figure 6B and Supplementary Figure S2A-S2B). Tumor latency and early tumor development did not vary between *PTEN*-KO and *PTEN/P53*-DKO mice, however, tumors from *PTEN/P53*-DKO showed significantly faster growth rates after 30 weeks of age that mirrored decreased survival (Figure 6B). Tumors from both models showed similar histological features during the early stages of development. However, as mice aged, tumors from *PTEN/P53 DKO* demonstrated faster progression to invasive adenocarcinomas and eventually transitioned from glandular differentiation to sarcomatoid histology (Figure 6C). Sarcomatoid transdifferentiation is associated with an epithelial to mesenchymal transition (EMT), a phenotypic event that leads to increased tumor invasion. In concert with this histological phenotype, *PTEN/P53*-DKO demonstrated a higher propensity to develop to distant metastases compared to *PTEN*-KO mice (Figure 7A-7B). Expression of phosphorylated AKT and AR was present in metastases but at reduced levels compared to primary tumors (Figure 7C). In summary, we have established a GEM model of prostate cancer in which the concomitant inactivation of *PTEN* and *P53* promotes the development of an aggressive phenotype that is characterized by accelerated tumor growth, increased invasion and development of metastatic disease that resembles late stage disease. These features make this an attractive system to further develop preclinical testing strategies for novel therapeutic strategies.

### AZD5363 monotherapy improves treatment outcomes in a mouse model of advanced prostate cancer

Thus far, our studies have shown that inhibition of AKT activity with AZD5363 produced favorable antitumor responses in models of *PTEN*-deficient early-stage CNPC and CRPC. Our next aim was to determine if targeted therapy with AZD5363 would result in improved treatment outcomes in a model of advanced prostate cancer. To achieve this goal, we emulated a randomized phase trial with AZD5363 in *PTEN/P53*-DKO mice (Supplementary Figure S1B). Mice with established disease, defined by bulk palpable tumor of 1 cm, were randomized to vehicle or AZD5363 as previously dosed. Study endpoints were overall survival, progression-free survival, tumor burden, and overall well-being based on performance score.

**Figure 8:**
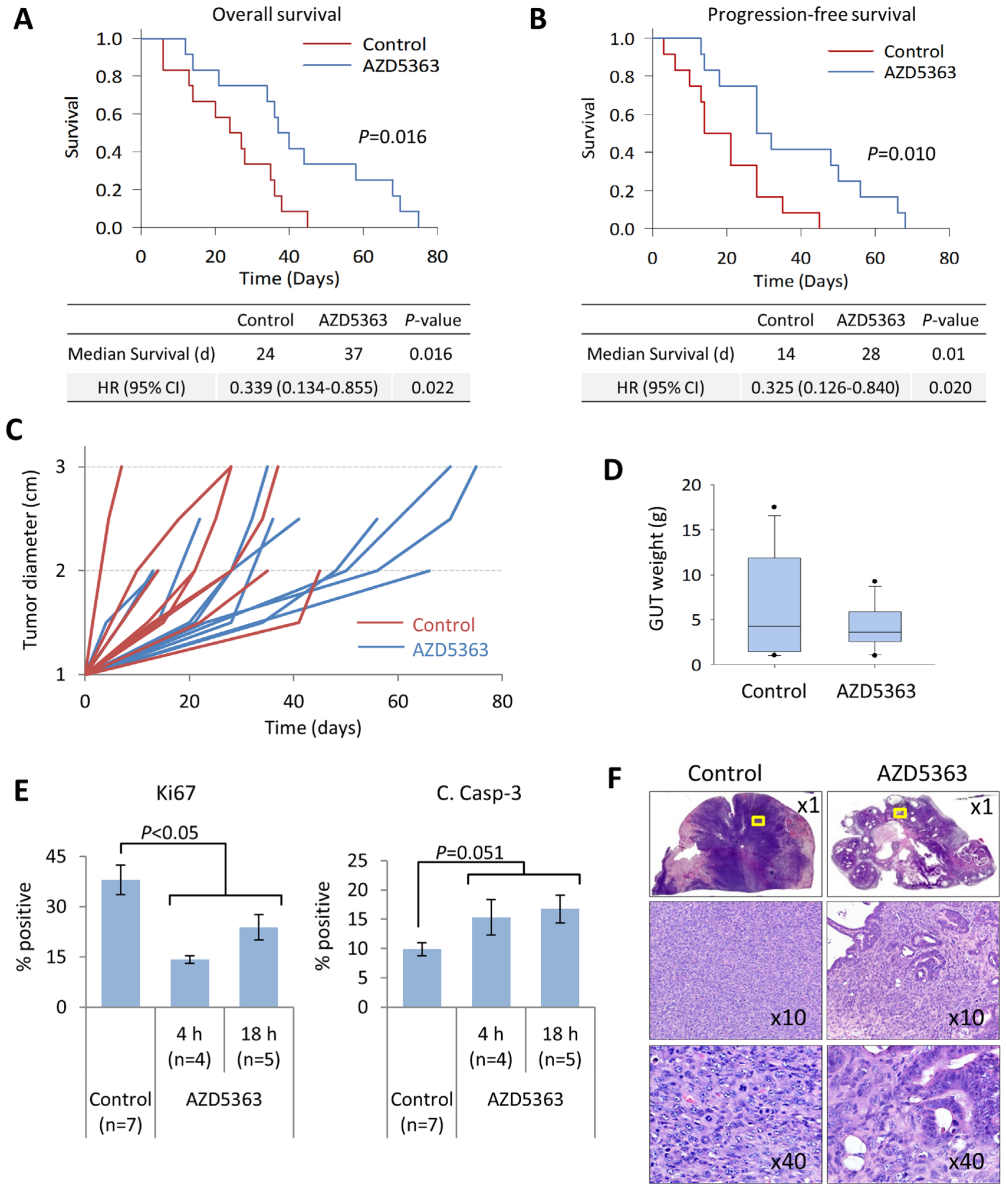
AZD5363 monotherapy delays tumor progression and improves survival in a model of advanced prostate cancer *PTEN/P53*-DKO mice were randomized (*n* = 12 per group) to control or AZD5363 (100 mg/kg, b.i.d) when palpable tumors reached 1 cm. Kaplan-Meier plots comparing overall survival **A.** and progression-free survival **B.** between control and AZD5363-treated mice. **C.** Quantification of prostate tumor growth rates, assessed by palpation. **D.** Box plots of tumor burden based on GUT weight from fresh tissues or post mortem tissues collected within 16 h of expiry. Boxes represent 25^th^-75^th^ quartiles, horizontal lines represent median vertical bars represent ± s.d., and dots represent minimum and maximum values. **E.**, IHC quantification of tumor proliferation and apoptosis rates between control and AZD5363-treated mice. Times correspond to the last administered dose of AZD5363. Values represent mean ± s.e. **F.**, Representative H&E stained sections of prostate tumor from control and AZD5363-treated mice. The x10 and x20 panels correspond to the yellow bounded regions.

A summary of the animal characteristics and treatment outcomes is shown in Table 1. There were no significant differences in the starting age, initial and final body weights between mice randomized to treatment with AZD5363 and control. Mice treated with AZD5363 demonstrated significant improvements in overall survival and progression-free survival compared to controls (Figure 8A-8B). Overall, primary tumors from mice receiving AZD5363 demonstrated decreased growth rates and tended to be smaller compared to control mice at *post mortem*, median GUT weight 3.6 *vs*. 4.3 g, *P* = 0.845, respectively (Figure 8C-8D).

**Table 1:** Mouse characteristics and treatment responses

Description	Control	AZD5363	*P*-value
No. of Mice	12	12	
Median age (w)	47.6	49.5	0.908
range	42.4-74.5	39.0-63.1	
Median initial bodyweight (g)	36.6	35.1	0.671
range	27.6-41.0	28.3-53.3	
Median final bodyweight (g)	34	37.2	0.353
range	27.8-49.1	29.9-43.3	
Median treatment duration (d)	25.5	38.5	**0.020**
range	6.0-45.0	12.0-75.0	
Median Age at Death (w)	51.7	55.2	0.299
range	43.8-80.0	45.3-69.0	
Median OS (d)	24.0	37.0	**0.016**
95% CI	12.1-35.9	30.2-43.8	
Median PFS (d)	14	28	**0.010**
95% CI	7.2-20.8	16.1-39.9	
Median GUT weight (g)	4.3	3.6	0.840
range	1.0-17.5	1.0-9.2	
Metastatic rate (%)	50	40	0.679
frequency	6/12	4/12	
Metastatic Burden (mean no. of mets/mouse)	2.5	2.5	1.00
s.e.	0.8	0.6	
Median time to progression P1-P2 (days)	21.0	28.0	0.110
range	3.0-45.0	13.0-66.0	
Median time to progression P2-P3 (days)	8.0	19.0	0.295
range	4.0-18	7.0-20	
Performance status (% favorable)	25	42	0.667
frequency	3/12	5/12	

We next performed IHC analyses to assess the effect of AZD5363 on proliferation and apoptosis in this setting. We collected tumor samples from mice selected for preemptive euthanasia. Mice were sacrificed approximately 2-4 h after the last treatment in in good condition or 12-18 h after the last treatment dose if in poor condition. Areas of viable tumor were evaluated for Ki67 and cleaved caspase-3 expression as before. Mice receiving AZD5363 demonstrated a significant decrease in the proliferation rates and a strong trend for increased apoptotic rates compared to control mice (Figure 8E). A greater degree of inhibition of tumor cell proliferation was observed in tissues from mice collected 4 h after last treatment dose compared to 18 h. No differences were observed in levels of cleaved caspase-3 expression between 4 and 18 h. Histological patterns of response varied between mice receiving AZD5363. However, in some cases, tumors from mice treated with AZD5363 demonstrated decreased sarcomatoid differentiation (Figure 8F).

**Figure 9:**
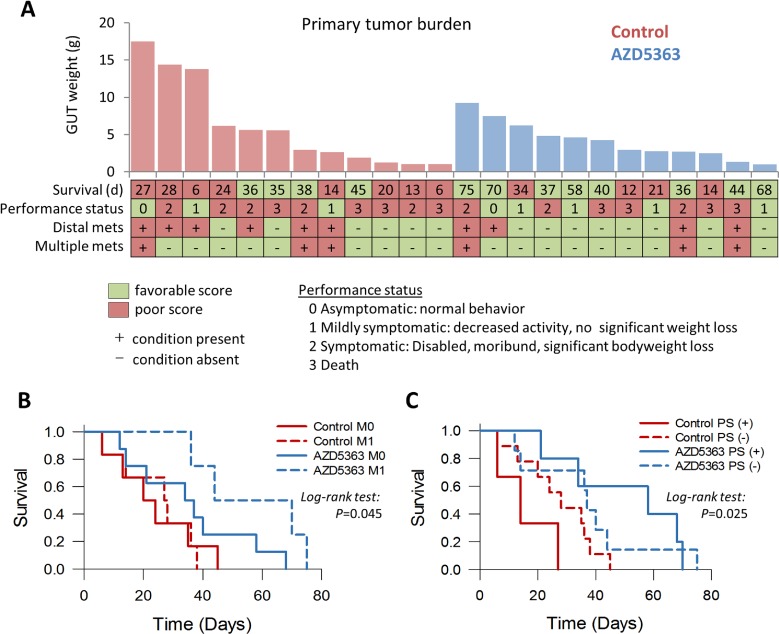
Influence of AZD5363 monotherapy in tumor progression and performance status in a late-stage model of mouse prostate cancer **A.**, Comparison of individual outcomes for *PTEN/P53*-DKO mice randomized to control or AZD5363 (M0, no metastasis present; M1, metastasis present; PS (+), favorable performance status; PS (−), poor performance status). Kaplan-Meier plots of overall survival for *PTEN/P53*-DKO control and AZD5363 treated *versus* the presence of metastasis **B.** and performance status **C.**.

Overall, treatment with AZD5363 did not appear to appreciably affect the development and spread of metastatic disease (Table 2). However, mice with metastatic disease that received treatment with AZD5363 tended to experience longer survival times compared to control mice with metastases (median survival 44 *vs*. 27 days respectively, *P* = 0.087, Figure 9A-9B). Performance status is an important assessment related to prognosis for patients with advanced cancer [24]. The performance status (PS) of mice used in this study was an attempt to quantify the overall well-being of tumor bearing mice receiving therapy. The scale was derived from the ECOG scoring system which runs from 0 to 5, in which a score of 0 denotes a healthy individual and a score of 5 indicates death [25]. The well-being of mice was judged as described in the Materials and Methods section and assessed a score ranging from 0 to 3 as follows: 0, asymptomatic; 1, mildly symptomatic; 2, symptomatic; 3, death. We assessed the effects of AZD5363 on morbidity by comparing mouse performance status. A favorable status was allocated to mice with a performance score ≤1, and a poor status to those with a score ≥2. Forty-two percent (5/12) of mice treated with AZD5363 had favorable status at euthanasia compared to 25% (3/12) in the control group (*P* = 0.667, Figure 9A). Furthermore, mice receiving AZD5363 with favorable performance status tended to experience longer survival times compared to AZD5363-treated mice poor with performance status or control mice with either favorable or poor performance scores (Figure 9C)

Collectively, our findings demonstrate that AKT inhibition is effective in inhibiting tumor growth and progression in various GEM models of *PTEN*-deficient prostate cancer. Thus, therapeutic strategies using potent AKT inhibitors such as AZD5363 may serve a possible treatment option for human prostate cancers with PI3K/AKT pathway alterations.

**Table 2:** Summary of metastatic involvement by site

	No. of mice	Location								Total
		Liver	Lung	Mes	Diaph	Panc	Spleen	Kidney	LN	
Control	6	2	2	4	0	0	1	3	3	15
AZD5363	4	1	3	1	2	2	0	0	1	10

Diaph, diaphragm; LN, lymph node; Mes, mesentery; Panc, pancreas

## DISCUSSION

In this study, we have presented a refined approach for preclinical drug screening using GEM models of *PTEN*-deficient prostate cancer. More significantly, we have applied this methodology to demonstrate the preclinical efficacy of AKT inhibition using AZD5363. Thus, providing lines of evidence to suggest that inhibition of AKT activity may provide beneficial therapeutic effects for patients suffering with prostate cancer with PI3K/AKT pathway alterations.

Several novel agents have been recently developed to target PI3K signaling and are in the early phases of clinical development [26]. AZD5363 is a potent pan-AKT inhibitor that has completed Phase 1 for solid tumors and is currently undergoing Phase 2 evaluation as a single agent or as combination therapy. In preclinical studies, AZD5363 has shown preferential sensitivity in models with PI3K pathway alterations (*PIK3CA, AKT1* and/or *PTEN* mutations) *in vitro* and *in vivo* [14-16, 27]. However, predicting the therapeutic potential through preclinical screening remains a challenge. We sought to predict the therapeutic efficacy of AZD5363 by first establishing a robust and clinically relevant model of prostate cancer which could be utilized to novel therapeutic agents.

A greater understanding of cancer molecular biology and the disease process has led to significant improvements being made in the development of anticancer therapies. Over time, the focus of anticancer drug development has shifted from conventional chemotherapy, relying mainly on cytotoxic agents, to target-oriented strategies. The last five decades have also shown an evolution in the use of mouse models for preclinical drug screening [28]. The ultimate goal of preclinical testing is to prospectively predict the clinical response, however, this has proven to be a major challenge using traditional *in vivo* mouse models [29]. An optimal preclinical animal model should utilize an approach that mirrors the human disease with well-defined endpoints that are clinically relevant. In addition, this approach should consider similar dosing schedules and routes of administration as those intended for humans.

Both GEM and PDX models have shown improvements over traditional cell based xenograft models with regards to drug predictability. However, as with any model, each has its own merits and limitations. PDX models tend to preserve the molecular diversity of the parental tumors and are better suited for the development personalized treatment strategies, however, the primary tissue source is limited and the process is technically complicated. There are also concerns with regards to the preservation genomic stability and the replacement of the human stroma with mouse stroma after serial transplantations. In addition, these models lack a competent immune system and bypass tumor initiation. GEM models are designed to mimic human cancers by altering the expression of one or more relevant genes and results in the generation tumor models that recapitulate most aspects of the tumorigenesis process [9]. Tumors develop in immunocompetent mice a natural environment. However, both tumor and the microenvironment are murine and accuracy of disease representation can vary. Overall, both GEM and PDX models provide excellent platforms to study new drugs but their application depends on context as well as technical and logistical circumstances. Ideally both models should complement each other. The use of GEM for preclinical testing has increased recently as models have become more widely available. However, considering the complexity of the disease process, it is highly implausible that any one model will be able to satisfy all of the desired requirements. We have addressed this issue by designing a preclinical screening paradigm that uses two GEM models of prostate cancer. The first model is used to evaluate drug activity after acute and chronic drug administration in the context of castration-naïve and castration-resistant disease while the second model is used to determine the treatment effects using clinically relevant endpoints.

The first model involves of the previously generated and characterized conditional *PTEN*-KO mouse model of prostate cancer [17]. Inactivation of *PTEN* has been thoroughly characterized a driver during prostate tumorigenesis in GEM [30]. Additionally, PI3K pathway activation has been reported in approximately half of primary tumors and is present in nearly all metastases as a result of *PTEN* loss of function [6, 31]. In our *PTEN*-KO model, inactivation of *PTEN* results in constitutively activate PI3K/AKT signaling which leads to the step-wise development of prostate tumor with complete penetrance and relatively short latency [17]. In addition *PTEN*-KO mice feature some characteristics of human prostate cancer such as the progression to CRPC after androgen withdrawal, and development of metastasis. We chose to use this model to perform initial pharmacodynamic analysis and to test the effects on tumor growth inhibition because mice consistently develop tumors containing mPIN lesions at a relatively early age (10-12 weeks) that demonstrate rapid growth from 12-25 weeks. At this age, untreated mice demonstrate little inter-tumor variability. We theorized that having a model with minimal inter-tumor variability would be desirable to confirm of drug activity and to establish an initial screen of drug tolerability and antitumor efficacy after chronic dosing. We tested this hypothesis by first confirming the inhibitory activity of AZD5363 in a pharmacodynamic experiment. This type of assessment can provide valuable *in vivo* pharmacodynamic data that establishes a biologically active dose that can then be used in the drug intervention experiments. Based on the results of the pharmacodynamic experiments, we established that a dose of 100 mg/kg b.i.d. would provide the desired inhibitory effect in our model. Indeed, chronic administration of AZD5363 at this dose and schedule proved to be effective in inhibiting AKT activity inducing tumor growth suppression in castration-naïve and castration resistant disease. One of our aims was to determine if chronic administration of AZD5363 would yield a therapeutic benefit based clinically relevant endpoints that measure survival, tumor burden, disease progression and performance status. Short latency and consistency in tumor development has made the *PTEN*-KO mouse model useful to screen for antitumor activity of novel agents and to characterize pertinent molecular mechanisms. At the same time, tumor growth is slow and mice tend to live long making this model impractical to conduct long-term studies. This quandary led us to develop the PTEN/P53 DKO mouse model.

*P53* is commonly associated with advanced metastatic prostate cancer and its influence on prostate cancer progression has been detailed [6, 22, 32]. As in *PTEN*-KO mice, inactivation of *P53* is also driven by the *PSA-Cre* promoter and represents a loss of function (null) mutation. Inactivation of *P53*, in the presence of *PTEN*, was clearly associated with increased tumor growth, histological progression to adenocarcinoma, development of distant metastasis and decreased survival. Notably, inactivation of *P53* alone did not lead to prostate cancer, meaning the concomitant inactivation of both *PTEN* and *P53* produces a model of cancer progression that follows the “multi-hit” hypothesis [33]. It is important to mention that a previous study reported similar observations when inducing the conditional inactivation of *PTEN* and/or *P53* and *PTEN/P53* in a mouse prostate cancer model using the *Probasin-Cr4* promoter [34]. However, in our study, mice with the biallelic inactivation of *PTEN/P53* experienced longer cumulative survival compared to the model previously reported, 12 months *vs*. 5 months, respectively [34]. The authors also noted that in their study, no mice survived past the age of 7 months and no distant metastases were noted. The difference in results could be attributed to the region of *P53* recombination and choice of promoter. In our model, the *PSA-Cre* promoter targeted exons 2-10 of *P53* in the luminal cells of the adult prostate, whereas, the authors used *Probasin-Cr4,* to target exon 7 in both luminal and basal cells of the developing and adult prostate [34-37].

Having established a mouse model of advanced prostate cancer, we designed a drug intervention trial mimicking a clinical trial design to measure the influence of AZD5363 therapy on clinically relevant endpoints. Our results clearly demonstrated that mice treated with AZD5363 experienced significant improvements in overall survival and progression-free survival despite the lack of a statistically significant reduction of tumor burden. In fact, this finding is similar to what is often experience with non-cytotoxic molecular targeting therapies [38, 39]. The term clinical benefit is used often to describe efficacy in clinical trials, although the definition is vague and tends to vary. In advanced prostate cancer, measurable disease can be difficult in a majority of cases. As a result, a shift in the focus from response to time-to-event end points has been recommended in for non-cytotoxic therapies [39]. The FDA considers overall survival and symptom endpoints as evidence for regulatory requirements of clinical effectiveness with several other endpoints serving as established surrogates including objective response rate and progression free survival among others [40]. Therefore it would make sense to include such parameters in preclinical testing. Other investigators have already applied this in *KRAS* mutant mouse models of non-small-cell lung carcinoma and pancreatic adenocarcinoma and in mouse “co-clinical trials” [41-45].

It is interesting to note that different mechanisms of response were observed between castration-naïve tumors and CRPC. In the castration-naïve prostate cancer models using either *PTEN*-KO or *PTEN/P53*-DKO mice, inhibition of AKT reduced tumor cell proliferation while inducing apoptosis, whereas, it appeared to induce a cytostatic response in CRPC. Our data provides evidence to support that a significant degree of crosstalk exists between the PI3K, MAPK and JAK/STAT3 signal transduction pathways. In the PD study, we revealed an induction of ERK phosphorylation after high-dose administration of AZD5363. Additionally, a transient increase in STAT3 phosphorylation was correlated to decreased AKT substrate activity. Our findings also showed that levels of phosphorylated ERK and STAT3 increased in proliferative tumor cells suggesting that inhibition of PI3K/AKT pathway activates MAPK and JAK/STA3 survival signaling that could eventually lead to therapeutic resistance. The activation of MAPK in response to PI3K pathway is commonly seen and could be the result of the induction of expression and phosphorylation of receptor tyrosine kinases modulated by the S6K/PI3K feedback loop [46]. In addition, JAK/STAT3 is commonly upregulated in CRPC; this may be mediated in part by PIM kinases, generating resistance to AKT inhibition [47]. Another possible mechanism mediating resistance could be attributed reciprocal feedback regulation between the androgen receptor (AR) and PI3K/AKT signaling networks [48, 49]. Further studies will need to be carried out to establish the precise mechanisms for decreased activity in CRPC, however, this study provides the critical data that can contribute to the develop rational combination therapies targeting multiple oncogenic pathways.

Our paradigm to assess preclinical drug efficacy has some limitations. Namely that our cancer models are based on mouse and have fundamental biological and physiological differences to humans. For example, sarcomatoid differentiation was frequently observed in our *PTEN/P53*-DKO model, even though, sarcomatoid cancers of the prostate are uncommon in humans. The progression to sarcomatoid differentiation is a feature quite common with various GEM models of invasive cancer, particularly those involving *PTEN* and *P53* gene inactivation [34, 50-53]. In our model, there was a heterogeneous adenocarcinoma-to-sarcomatoid progression that was characterized by increased metastasis, presumably indicating that sarcomatoid arose as a result of EMT. In human prostate cancer, hormone sensitive cancers typically start out as adenocarcinoma that has an epithelial structure, however, during disease progression, histopathological changes from differentiated to undifferentiated, i.e., structural to nonstructural may occur in a majority of cases. This is the natural history of human prostate cancer. The purpose of the *PTEN/P53*-DKO mouse model was to establish a more aggressive phenotype that results in metastasis, disseminated disease. In this regard, the natural history of the *PTEN/P53*-DKO mouse is similar to that of human prostate cancer with a higher malignant potential. We also opted to use palpation as a means to assess tumor burden in *PTEN/P53*-DKO mice. Although this method is subjective, it does provide a rapid method to monitor tumor burden with minimal stress to mice. Using imaging techniques requires a significant amount of time, and regular assessments of tumor burden would have subjected the mice to additional exposure to anesthesia and undue strain. Nevertheless, we believe that this paradigm can provide essential information that can be used with other models of preclinical efficacy to improve predictability of novel treatment strategies.

In summation, our study provides preclinical evidence to support targeting of PI3K signaling through AKT inhibition in *PTEN*-deficient prostate cancer. In addition, we presented an approach for robust preclinical evaluation of novel drug efficacy using relevant GEM models of the disease.

## MATERIALS AND METHODS

### Drugs and reagents

AZD5363 was synthesized by AstraZeneca, and solubilized in a 10% DMSO, 25% w/v KLEPTOSE (Roquette) solution. Primary antibodies used for this study are listed in Supplementary Table S1.

### Animals

*PSA-Cre* and *PSA^Cre^;PTEN^loxP/loxP^* (*PTEN* KO) mice have been previously described [17, 54]. *P53* floxed mice (01XC2) were obtained through the NCI Mouse Repository [35]. *P53* floxed mice were bred with *PSA-Cre* mice and backcrossed to produce *PSA^Cre^;P53^loxP/loxP^* offspring. *PSA^Cre^;P53^loxP/loxP^* mice were bred with *PSA^Cre^;PTEN^loxP/loxP^* mice and backcrossed to produce *PSA^Cre^;PTEN^loxP/loxP^;P53^loxP/loxP^* double knockout (*PTEN/P53-D*KO) offspring. To produce CRPC, 10-12-week-old mice were surgically castrated as previously described [17]. Mice were housed at Kinki University Faculty of Medicine Animal Facility in accordance with institutional guidelines and procedures were carried out in compliance with the standards for use of laboratory animals. This study was approved by the Institutional Review Committee at Kinki University Faculty of Medicine.

### PCR Genotyping

Genotyping for the *Cre-recombinase, PTEN* and *P53* was performed by PCR using tail biopsy DNA. DNA was extracted using the alkaline extraction method as previously described [17]. PCR primers and conditions are listed in Supplementary Table S3.

### *In vivo* drug testing

Pharmacodynamic experiments were performed on 20-week-old *PTEN*-KO mice harboring castration-naïve prostate cancer (CNPC). AZD5363 was administered orally as a single dose in dose- and time-dependent manner. Prostate tumors were dissected and processed for western blot analysis.

Drug efficacy studies were performed on 16-week-old homozygous *PTEN*-KO mice harboring CNPC or CRPC as previously described [17]. Mice were randomized to vehicle or AZD5363 (100 mg/kg, b.i.d.) for 4 weeks (Supplementary Figure S1A). Mice were euthanized 2 h after the last treatment dose and genitourinary tracts (GUT) were removed, weighed and imaged. Half of the prostate gland was removed and flash frozen in LN_2_ and stored at −80°C for the collection of protein. The remaining portion was fixed overnight in 10% neutral buffered formalin, processed and embedded in paraffin for further analysis. Tumor burden was determined by prostate surface area as previously described [17].

A survival efficacy trial was performed using *PTEN/P53*-DKO mice developed in our laboratory. Mice were palpated weekly beginning at 35 weeks of age and were randomized to treatment once prostate tumors reached 1 cm in diameter (Supplementary Figure S1B). Mice were treated with vehicle or AZD5363 (100 mg/kg, b.i.d.) until either of the following criteria occurred: expiry, tumor diameter > 3 cm, > 20% bodyweight loss from baseline, > 10% bodyweight loss/week or poor performance status, in which case mice were euthanized. Study endpoints were overall survival, progression-free survival, tumor burden, and performance status. Disease progression was defined as expiry, tumor size increase to > 2 cm in diameter, > 20% bodyweight loss from baseline, > 10% bodyweight loss/week and poor performance status. The performance status representing the overall well-being of mice was determined by trained animal staff and assessed a score on the basis of bodyweight changes, physical appearance, level of activity, behavior and demeanor. Based on these criteria, a score for the worst case scenario was assigned as follows: a score of 0 represents a healthy animal; a score of 1 represents a mouse that exhibits reduced levels of behavioral activity (burrowing, grooming) but still exhibits good mobility and tendency to scape, slight body bodyweight weight loss (< 10% from baseline) and normal defecation/urination; a score of 2 represents a mouse with any of the following: sick appearance (hunched posture, sluggish movements, no escape reflex), stressed (not eating or drinking, labored or fast breathing), significant bodyweight loss (> 10% bodyweight loss in one week or > 20% bodyweight loss from baseline), or the presence of diarrhea or hematuria. All mice had a score of 0 at randomization. Tumor burden was determined by primary tumor weight and the number of metastatic lesions.

### Histology and immunohistochemistry

For histological analysis, slides were stained in hematoxylin and eosin (H&E). Histopathological classification of mouse prostate lesions was performed by trained research staff according to the criteria proposed by the Bar Harbor Classification system [53]. Distribution analysis of tumor gland differentiation was performed as previously described [21]. For immunohistochemistry (IHC), additional slides were incubated sectioned and stained using the ABC kit (Vector Laboratories) following manufacturer's protocols. Antibodies and specific pretreatments are listed in Supplementary Table S4. Assessment of IHC staining was performed as previously described [21].

### Western blot analysis

Protein extraction was performed in RIPA buffer with HALT protease and phosphatase inhibitors (Thermo Scientific). SDS-gel electrophoresis, western blotting and semi-quantitative densitometric analyses using ImageJ were performed as previously described [17].

### Statistical analysis

The Student's *t*-test for was used to calculate two-tailed significance of paired analysis, one-way ANOVA for multiple comparisons, *chi*-square test for proportions and polynomial regression analysis for tumor growth dynamics. Kaplan-Meier survival curves using the log-rank test were used to measure survival. Differences were considered to be significant at *P* < 0.05. Statistical analysis was carried out using SigmaPlot v.13.0 (Systat Software).

## SUPPLEMENTARY MATERIAL FIGURES AND TABLES


