# Supplementary material for: Efficacy of targeted AKT inhibition in genetically engineered mouse models of PTEN-deficient prostate cancer
Source: Oncotarget. 2016 Feb 21;7(13):15959–76. doi: 10.18632/oncotarget.7557 (PMC4941290; doi:10.18632/oncotarget.7557)
Supplement: Supplementary file 1 [file oncotarget-07-15959-s001.pdf]

Efficacy of targeted AKT inhibition in genetically engineered mouse models of *PTEN*-deficient prostate cancer

Supplementary Material

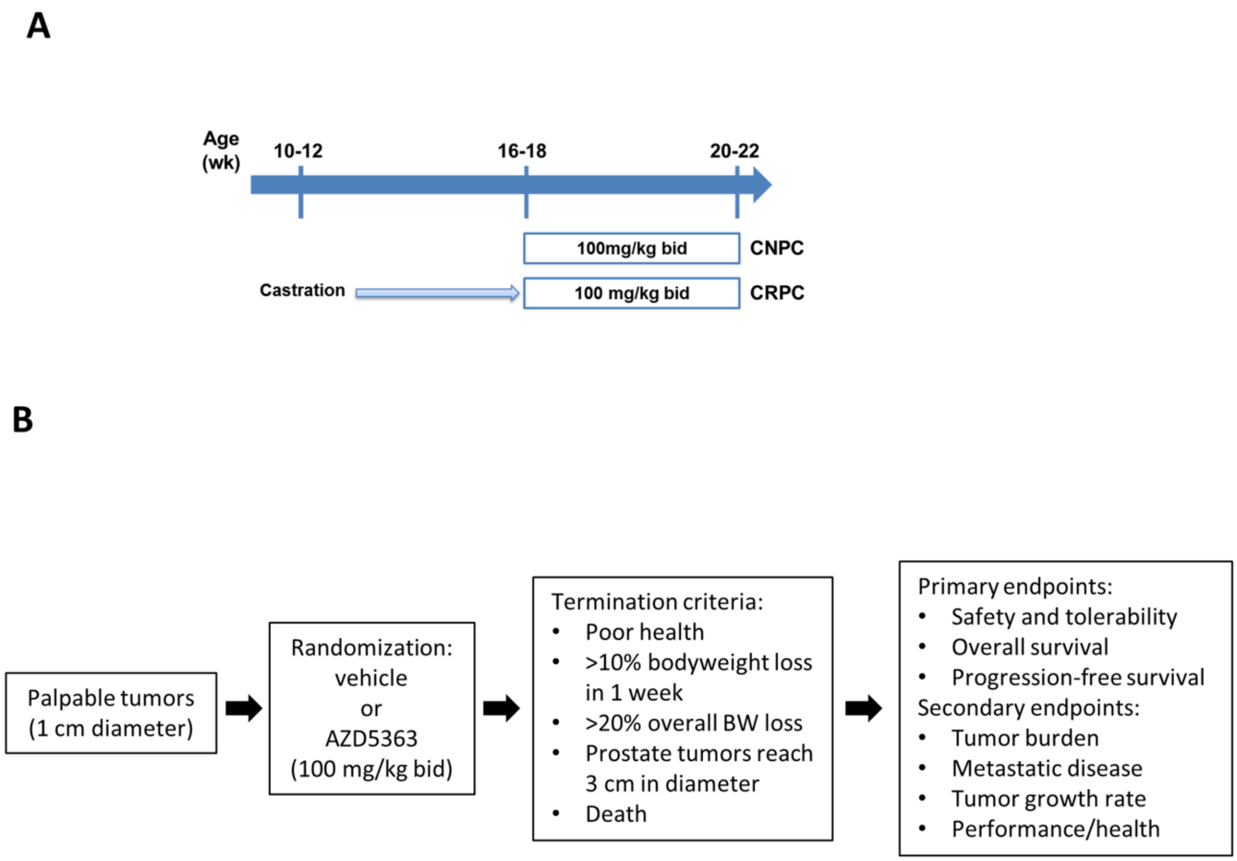

Supplementary Figure S1. A. The experimental design for a drug efficacy screen using *PTEN*-KO mice that harbor castration-naïve or castration resistant prostate tumors. **B.** Experimental design for late-stage disease using *PTEN/P53*-DKO mice.

A

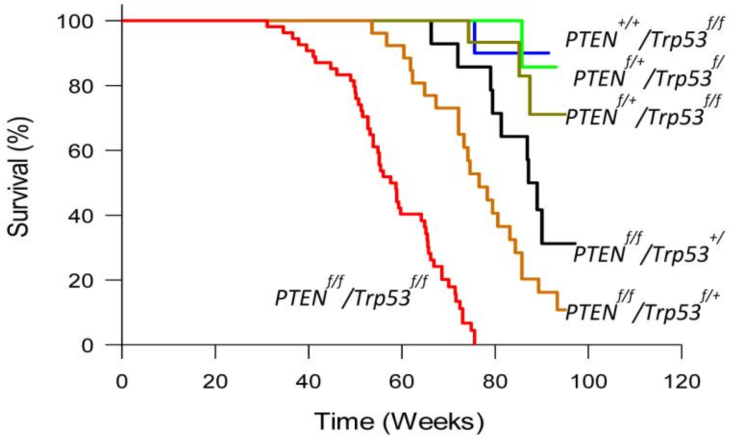

B

Pairwise multiple comparisons for survival in conditional *PTEN and PTEN/Trp53* knockout mice

Comparisons	Statistic	Unadjusted P Value	Critical Level	Significant
PTEN$^{f/+}$/*Trp53*$^{f/f}$ vs. *PTEN*$^{f/f}$/*Trp53*$^{f/f}$	39.904	2.67E-10	0.00341	**Yes**
PTEN$^{f/f}$/*Trp53*$^{+/+}$ vs. *PTEN*$^{f/f}$/*Trp53*$^{f/f}$	34.845	3.57E-09	0.00366	**Yes**
PTEN$^{+/+}$/*Trp53*$^{f/f}$ vs. *PTEN*$^{f/f}$/*Trp53*$^{f/f}$	34.703	3.84E-09	0.00394	**Yes**
PTEN$^{f/f}$/*Trp53*$^{f/+}$ vs. *PTEN*$^{f/f}$/*Trp53*$^{f/f}$	31.937	1.59E-08	0.00427	**Yes**
PTEN$^{f/+}$/*Trp53*$^{f/+}$ vs. *PTEN*$^{f/f}$/*Trp53*$^{f/f}$	22.465	0.00000214	0.00465	**Yes**
PTEN$^{f/+}$/*Trp53*$^{f/f}$ vs. *PTEN*$^{f/f}$/*Trp53*$^{f/+}$	12.131	0.000496	0.00512	**Yes**
PTEN$^{+/+}$/*Trp53*$^{f/f}$ vs. *PTEN*$^{f/f}$/*Trp53*$^{f/+}$	11.64	0.000646	0.00568	**Yes**
PTEN$^{f/+}$/*Trp53*$^{f/+}$ vs. *PTEN*$^{f/f}$/*Trp53*$^{f/+}$	9.569	0.00198	0.00639	**Yes**
PTEN$^{f/f}$/*Trp53*$^{+/+}$ vs. *PTEN*$^{f/f}$/*Trp53*$^{f/+}$	4.1	0.0429	0.0073	No
PTEN$^{f/f}$/*Trp53*$^{+/+}$ vs. *PTEN*$^{+/+}$/*Trp53*$^{f/f}$	3.892	0.0485	0.00851	No
PTEN$^{f/f}$/*Trp53*$^{+/+}$ vs. *PTEN*$^{f/+}$/*Trp53*$^{f/+}$	3.453	0.0631	0.0102	No
PTEN$^{f/f}$/*Trp53*$^{+/+}$ vs. *PTEN*$^{f/+}$/*Trp53*$^{f/f}$	2.441	0.118	0.0127	No
PTEN$^{+/+}$/*Trp53*$^{f/f}$ vs. *PTEN*$^{f/+}$/*Trp53*$^{f/f}$	0.544	0.461	0.017	No
PTEN$^{f/+}$/*Trp53*$^{f/+}$ vs. *PTEN*$^{f/+}$/*Trp53*$^{f/f}$	0.487	0.485	0.0253	No
PTEN$^{+/+}$/*Trp53*$^{f/f}$ vs. *PTEN*$^{f/+}$/*Trp53*$^{f/+}$	0.00489	0.944	0.05	No

The Holm-Sidak method was used for all pairwise multiple comparisons.

Supplementary Figure S2. *P53* inactivation promotes lethality in mouse *PTEN*-deficient prostate cancer. **A.** Kaplan-Meier plots comparing cumulative survival between combinations of monoallelic and biallelic inactivation of *PTEN* and *P53* mediated by *PSA-Cre*. **B.** Pairwise statistical comparisons of **A.**

Supplementary Table S1. List of PCR primers used for genotyping

Name	Sequence	Product sizes (bp)
PSA-Cre	sense (5' to 3')<br>GCCTATATCCCAAAGGAACAGAAG	330
	antisense (5' to 3')<br>CCTTCCTCTAGGTCCTTTAGGAGG	
PTEN	sense (5' to 3')<br>CTCCTCTACTCCATTCTTCCC	5' LoxP: 335<br>Wildtype: 228
	antisense (5' to 3')<br>ACTCCCACCAATGAACAAAC	
TrP53	sense (5' to 3')<br>CACAAAAACAGGTTAAACCCA	5' LoxP: 370<br>Wildtype: 288
	antisense (5' to 3')<br>AGCACATAG GAGGCAGAGAC	

PCR reaction conditions were as follows: initial denaturation at 94°C for 1 min, 35-cycle amplification at 94 C for 25 s, 60 C for 30 s and 72 C for 45 s, followed by final extension at 72 C for 10 additional minutes.

Supplementary Table S2. List of antibodies used for western blot and IHC

Name	Manufacturer	Cat#	Source	Predicated Band size	Dilution for Western Blot	Dilution for IHC	Antigen retrieval method
Akt	Cell Signaling	9272	Rabbit	60	1:1000	-	-
p-Akt	Cell Signaling	4060	Rabbit	60	1;1000	1:100	10mM Citrate buffer
Androgen receptor	Thermo	RB-9030	Rabbit	-	-		
Cleaved caspase-3	Cell Signaling	9661	Rabbit	17,19	1:1000	1:2000	Dako A.R.S
4E-BP1	Cell Signaling	9452	Rabbit	15-20	1:1000	-	-
p-4E-BP1	Cell Signaling	2855	Rabbit	15-20	1:1000	-	-
Erk	Cell Signaling	9102	Rabbit	42,44	1;2000	-	-
p-Erk	Cell Signaling	4370	Rabbit	42,44	1;2000	1:100	Dako A.R.S
FOXO1	Cell Signaling	2880	Rabbit	72-82	1:1000	-	-
p-FOXO1 (Ser256)	Cell Signaling	9461	Rabbit	82	1;1000	-	-
GAPDH	Cell Signaling	2118	Rabbit	37	1;25000	-	-
GSK-3β	Cell Signaling	9315	Rabbit	46	1:1000	-	-
p-GSK-3β	Cell Signaling	9336	Rabbit	46	1:1000	-	-
Ki67	Thermo	RB-9043	Rabbit	-	-	1:100	Dako A.R.S
S6	Cell Signaling	2217	Rabbit	32	1;2000	-	-
p-S6	Cell Signaling	2211	Rabbit	32	1;2000	1:100	Dako A.R.S
PCNA	Thermo	RB-9055	Rabbit	36	1;1000	-	-
Stat3	Cell Signaling	4904	Rabbit	79,86	1;1000	-	-
p-Stat3 (Tyr705)	Cell Signaling	9145	Rabbit	79,86	1;1000	1:100	Dako A.R.S
p-Stat3 (SerY727)	Cell Signaling	9134	Rabbit	79,86	1;1000	-	-

A.R.S., antigen retrieval solution; IHC, immunohistochemistry
